# Upregulation of HSP90α in the lungs and circulation in sarcoidosis

**DOI:** 10.3389/fmed.2025.1532437

**Published:** 2025-01-15

**Authors:** Takuma Isshiki, Motoko Sunakawa, Megan Vierhout, Anmar Ayoub, Pareesa Ali, Safaa Naiel, Shion Miyoshi, Asghar Naqvi, Nathan Hambly, Kazuma Kishi, Kjetil Ask, Martin R. J. Kolb

**Affiliations:** ^1^Department of Medicine, Firestone Institute for Respiratory Health, McMaster University, Hamilton, ON, Canada; ^2^Department of Pathology and Molecular Medicine, McMaster Immunology Research Center, McMaster University, Hamilton, ON, Canada; ^3^Department of Respiratory Medicine, Toho University School of Medicine, Tokyo, Japan

**Keywords:** sarcoidosis, granuloma, Hsp90α, extracellular Hsp90α, biomarker

## Abstract

**Background:**

Sarcoidosis is a systemic granulomatous disease of unknown cause. Natural improvement with favorable outcome is common, but a significant number of patients present with difficult to manage and progressive disease. The identification of biomarkers associated with disease activity and progression is warranted. Extracellular heat shock protein 90 (HSP90) *α* is a signaling molecule released by cells that induces proinflammatory signaling through interaction with certain receptors, such as lipoprotein receptor–related protein 1.

**Materials and methods:**

HSP90α protein expression in lung tissues derived from patients diagnosed with sarcoidosis and control subjects was assessed by immunohistochemistry. Serum HSP90α concentration was measured in sarcoidosis patients and healthy controls and correlated with clinical outcomes. Bronchoalveolar lavage fluid (BALF) was collected and analyzed for HSP90α expression. Extracellular HSP90α released from macrophages was examined in human primary cells and an immortalized cell line.

**Results:**

Macrophages and granulomas in sarcoidosis-affected lungs showed high HSP90α expression. Serum HSP90α levels were elevated in sarcoidosis patients compared with controls and correlated with BALF HSP90α levels. HSP90α concentrations in the circulation were correlated with biomarkers of disease stage. Both primary and immortalized macrophages showed a high capacity for secreting extracellular HSP90α.

**Conclusion:**

These results demonstrate that macrophages in the lungs of sarcoidosis patients produce high levels of HSP90α, suggesting HSP90α as a potential biomarker and therapeutic target.

## Introduction

Sarcoidosis is a systemic inflammatory disorder characterized by granulomatous inflammation in multiple organs, such as the eyes, lungs, skin, and lymph nodes (1). Structurally, these granulomas are well-formed and typically non-necrotizing, which cause damage that can ultimately lead to organ dysfunction.

Several biomarkers are clinically available for both diagnostic purposes and for evaluating disease activity. Angiotensin-converting enzyme (ACE) and soluble interleukin-2 receptor (sIL-2R) are associated with lung function in sarcoidosis but of limited practical use (2). Lysozyme is another marker that may reflect lymphocytic activation of the disease (3). Many efforts to identify diagnostic biomarkers that enable more accurate prediction of disease status are currently underway. Heat shock protein 90 (HSP90) *α* is an isoform of HSP90, which is an intracellular molecular chaperone protein present in a variety of cell types (4). The function of secreted extracellular HSP90 (eHSP90) *α* is distinct from that of the intracellular form, and eHSP90α has been shown to interact with receptors such as lipoprotein receptor–related protein (LRP1) and human epidermal growth factor receptor-2. Interaction between eHSP90α and LRP1 promotes downstream signaling via phosphorylation of STAT3, ERK1/2, PI3K, and AKT1/2, resulting in an upregulation of proinflammatory signaling (5–7). Recent studies have suggested that eHSP90α may be a useful marker for the diagnosis of inflammation and fibrotic diseases (8–10). Whether there is an association between sarcoidosis and HSP90α has not yet been determined.

In the present study, we examined the expression of HSP90α in the lungs and circulation of sarcoidosis patients. HSP90α is highly expressed in lung macrophages and granulomas of sarcoidosis. We found that macrophages are a major source of eHSP90α and that production of eHSP90α by these cells is upregulated further in response to cytokine stimulation. Sarcoidosis patients showed elevated eHSP90α levels in the circulation and lungs, which might be associated with the pathogenesis and progression of the disease.

## Materials and methods

### Patients

Forty sarcoidosis patients treated at Toho University Omori Medical Center, and 30 age- and sex-matched healthy controls were recruited for evaluation of eHSP90α. The study was approved by the Ethics Committee of Toho University School of Medicine (protocol number A22080). All study subjects provided written informed consent for participation.

Diagnosis was based on the American Thoracic Society/European Respiratory Society/World Association for Sarcoidosis and Other Granulomatous Disorders statement on sarcoidosis (11). Briefly, patients with histological findings of noncaseating epithelioid granulomas from tissue specimens with relevant clinical and radiologic findings were diagnosed as having sarcoidosis. ACE, sIL-2R, and lysozyme levels were measured in the clinical laboratory. Radiologic staging of lung lesions was determined based on chest radiography and computed tomography (12).

### Serum and bronchoalveolar lavage fluid (BALF) collection

Serum was collected at the time patients were enrolled in the study. Patients newly diagnosed with sarcoidosis during the study period underwent bronchoalveolar lavage with a fiberoptic bronchoscope. A total of 50 mL of saline was administered three times to the right medial lobe or left lingular lobe, and BALF was collected after each instillation. Collected serum and BALF were centrifuged at 3000 rpm for 10 min, and the resulting supernatants were aliquoted and frozen at −80°C until analysis.

### Enzyme-linked immunosorbent assay (ELISA)

The concentration of eHSP90α in patient serum and BALF and in the supernatant of medium was determined using a human HSP90α ELISA kit (Enzo Life Sciences, NY, USA) according to the manufacturer’s instructions.

### Human lung tissues

Formalin-fixed, paraffin-embedded human lung tissues from sarcoidosis and control lungs were obtained from the Biobank for Interstitial Lung Diseases at St Joseph’s Healthcare in Hamilton, Ontario, Canada. All work conducted using human tissues was approved by the Hamilton Integrated Research Ethics Board (11–3,559 and 13,523-C). Affected lung lesions of sarcoidosis patients and non-tumor areas of lung tissues from control subjects were selected and placed in a tissue microarray (TMA) block using TMA Master II (3DHISTECH Ltd., Hungary).

TMA slides were stained with hematoxylin and eosin (H&E), anti-CD68 antibody (Agilent Dako M0876, CA, USA), and anti-HSP90α antibody (Novus Biologicals NBP1-77685, ON, Canada). High-definition images were acquired using an Olympus VS120 Slide Scanner.

### Cell preparation

THP-1 cells were purchased from the American Type Culture Collection (ATCC#TIB-202). THP-1 cells and monocytes were differentiated into macrophages by treatment with phorbol myristate acetate (Millipore Sigma, ON, Canada) at 10 ng/mL for 48 h.

Human monocyte-derived macrophages (MDMs) were generated from three healthy donors. Peripheral blood mononuclear cells (PBMCs) were isolated from peripheral blood and purified by density-gradient centrifugation using BD vacutainer mononuclear cell preparation tubes (Becton Dickinson and Company, NJ, USA). CD14-positive cells were magnetically isolated from PBMCs using an Easy sep Human CD14-positive selection kit (STEMCELL Technologies, BC, Canada) according to the manufacturer’s protocol. CD14-positive cells were differentiated into unpolarized macrophages by treatment with 20 ng/mL macrophage colony-stimulating factor (Peprotech, QC, Canada) for 6 days.

### Cell culture and macrophage polarization

Macrophages were cultured in RPMI-1640 medium supplemented with 2 mM L-glutamine, 1% penicillin/streptomycin, and 10% fetal bovine serum. Macrophages were polarized toward the M1 phenotype by treatment with 100 ng/mL lipopolysaccharide (Peprotech, QC, Canada) and 20 ng/mL recombinant human interferon (IFN)-*γ* (Peprotech QC, Canada). The supernatant was collected at each time point and analyzed for eHSP90α concentration.

### Statistical analysis

Data are presented as mean ± SD. The Student’s *t* test or *χ^2^* test were used to compare differences between two groups. Receiver operating characteristic (ROC) curve analysis was conducted to determine the optimal cut-off value of serum eHSP90α. Analyses of correlations between two groups were performed using Pearson’s correlation test. A *p* value of <0.05 was considered to indicate statistical significance. All statistical analyses were carried out using GraphPad Prism, version 8 (MDF Co., Ltd., CA, USA).

## Results

### Expression of HSP90α in human lung tissues

**Figure 1:**
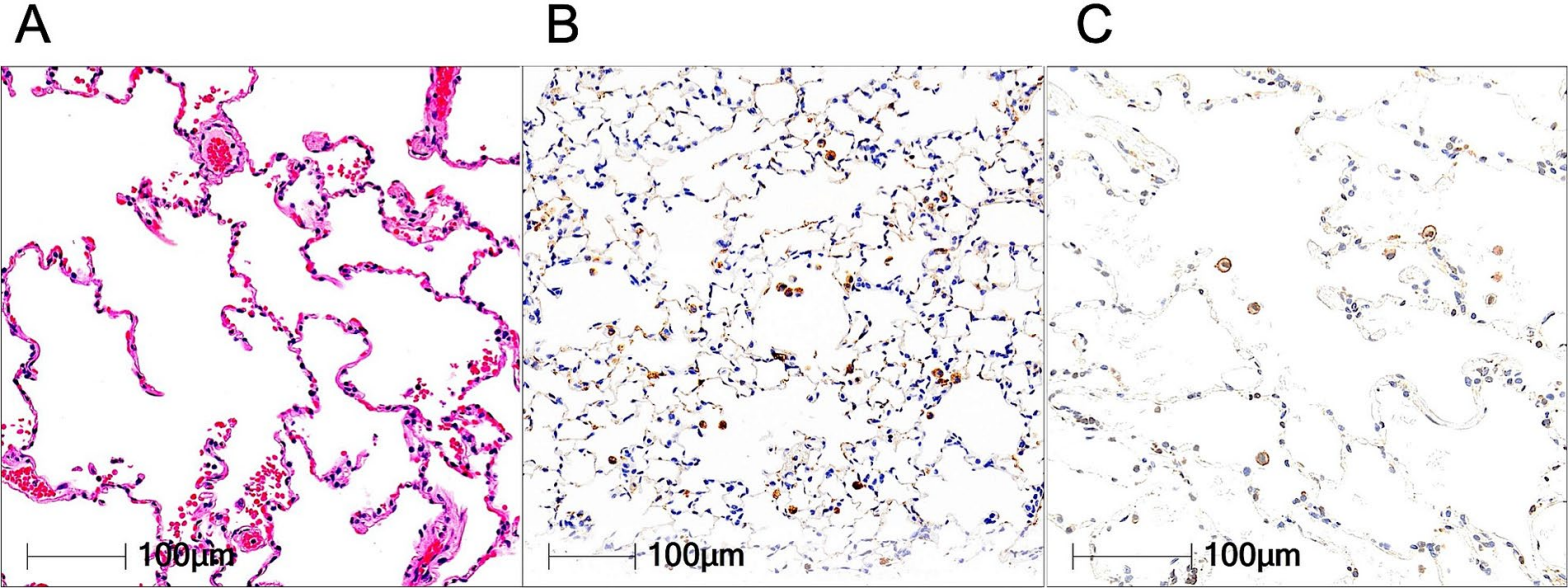
Expression of HSP90α in lungs as determined by immunohistochemistry. H&E staining **(A)**, CD68 staining **(B)**, and HSP90α staining **(C)** of normal lungs (*n* = 8). Positive signals were observed in lung macrophages.

We examined the expression of HSP90α by immunohistochemistry on TMA slide generated from 34 sarcoidosis lung cores and 8 control lung cores. Control lung tissues displayed strong expression of HSP90α by lung macrophages and some alveolar epithelial cells (Figure 1).

**Figure 2:**
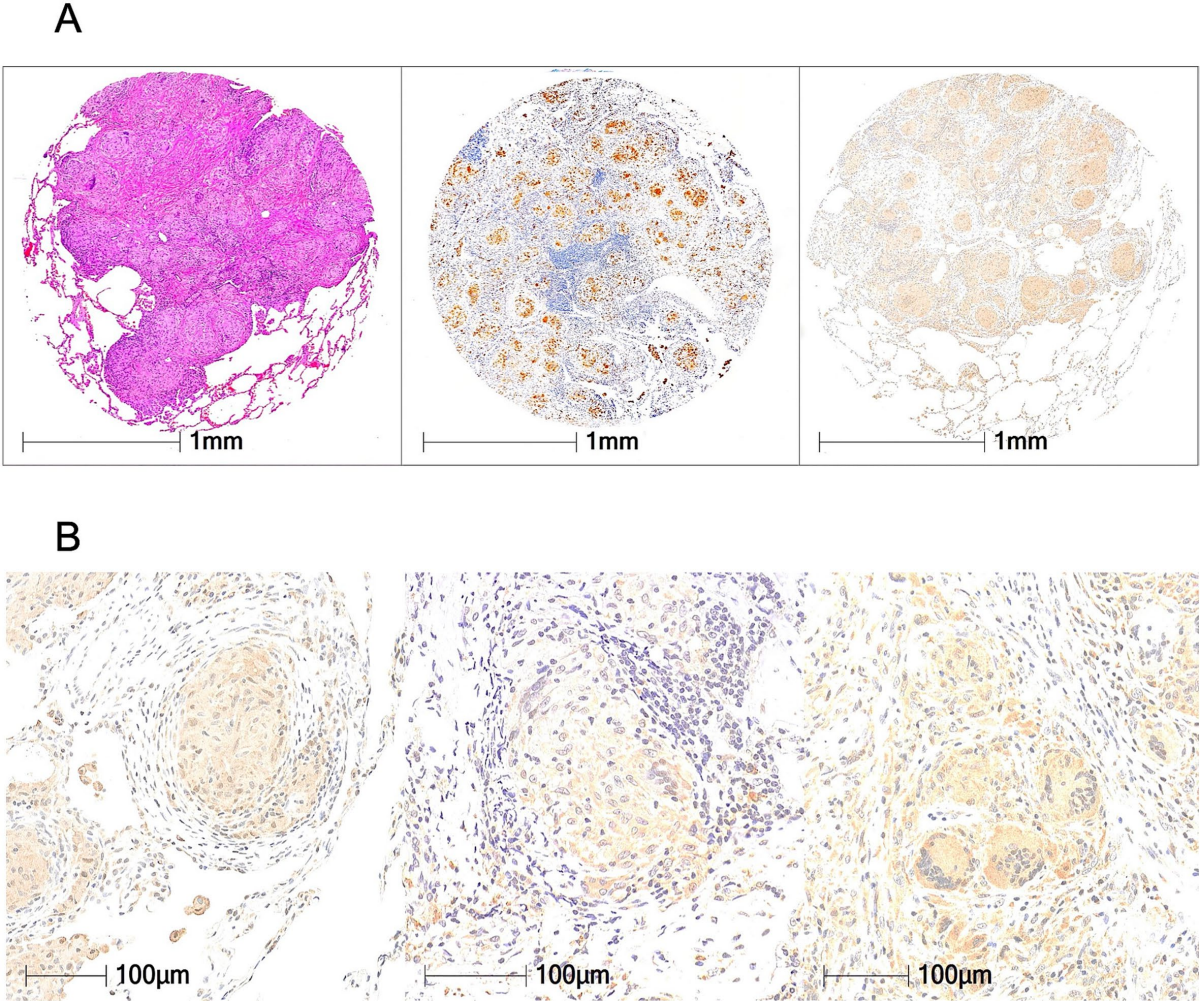
HSP90α expression in sarcoidosis lungs. **(A)** H&E staining (left), CD68 staining (middle), and HSP90α staining (right) of lung tissues of sarcoidosis from TMA slides (*n* = 34). **(B)** High-magnification image of sarcoidosis lungs stained for HSP90α. HSP90α was strongly expressed in the center of each granuloma.

Figure 2 shows representative TMA core sections of sarcoidosis lung tissues. Lung core regions containing granulomas were selected and isolated from lung biopsy specimens of 12 sarcoidosis patients under instruction of a lung pathologist (Supplementary Figure S1). Multiple epithelioid granulomas were observed upon H&E staining (Figure 2A). Granulomas showed overall highly positive signals for CD68, a pan macrophages marker, and HSP90α in the center region of granulomas (Figure 2A). Magnified images revealed that HSP90α expression was prominent in the central epithelial cells and multinucleated giant cells of granulomas but scarce in the interstitial cells and inflammatory lymphocytes surrounding the granuloma (Figure 2B).

### Serum eHSP90α levels in sarcoidosis

Based on the finding that HSP90α was strongly expressed in sarcoid granulomas, we hypothesized that eHSP90α may be upregulated in the circulation and at local sites of disease. To test this hypothesis, we determined the eHSP90α levels in serum and BALF of sarcoidosis patients. Forty sarcoidosis patients and 30 healthy sex- and age-matched individuals were included in the study. Baseline characteristics of the patients and healthy controls are described in Table 1.

**Table 1:** Baseline characteristics of sarcoidosis patients and healthy controls.

	Sarcoidosis (*n* = 40)	Healthy controls (*n* = 30)
Age (years)	59 ± 14	51 ± 11
Male, *n* (%)	20 (50%)	12 (40%)
Smoking history, *n* (%)	21 (53%)	9 (30%)
ACE (U/L)	20.5 ± 19.5	-
sIL-2R (U/mL)	666 ± 382	-
Chest stage (I/II/III/IV)	12/21/3/4	-
BALF CD4/8 (ratio)	4.5 ± 4.5	-
BALF lymphocytes (%)	32 ± 24	-

ACE, angiotensin-converting enzyme; sIL-2R, soluble IL-2 receptor; FVC, forced vital capacity; BALF, bronchoalveolar lavage fluid.

**Figure 3:**
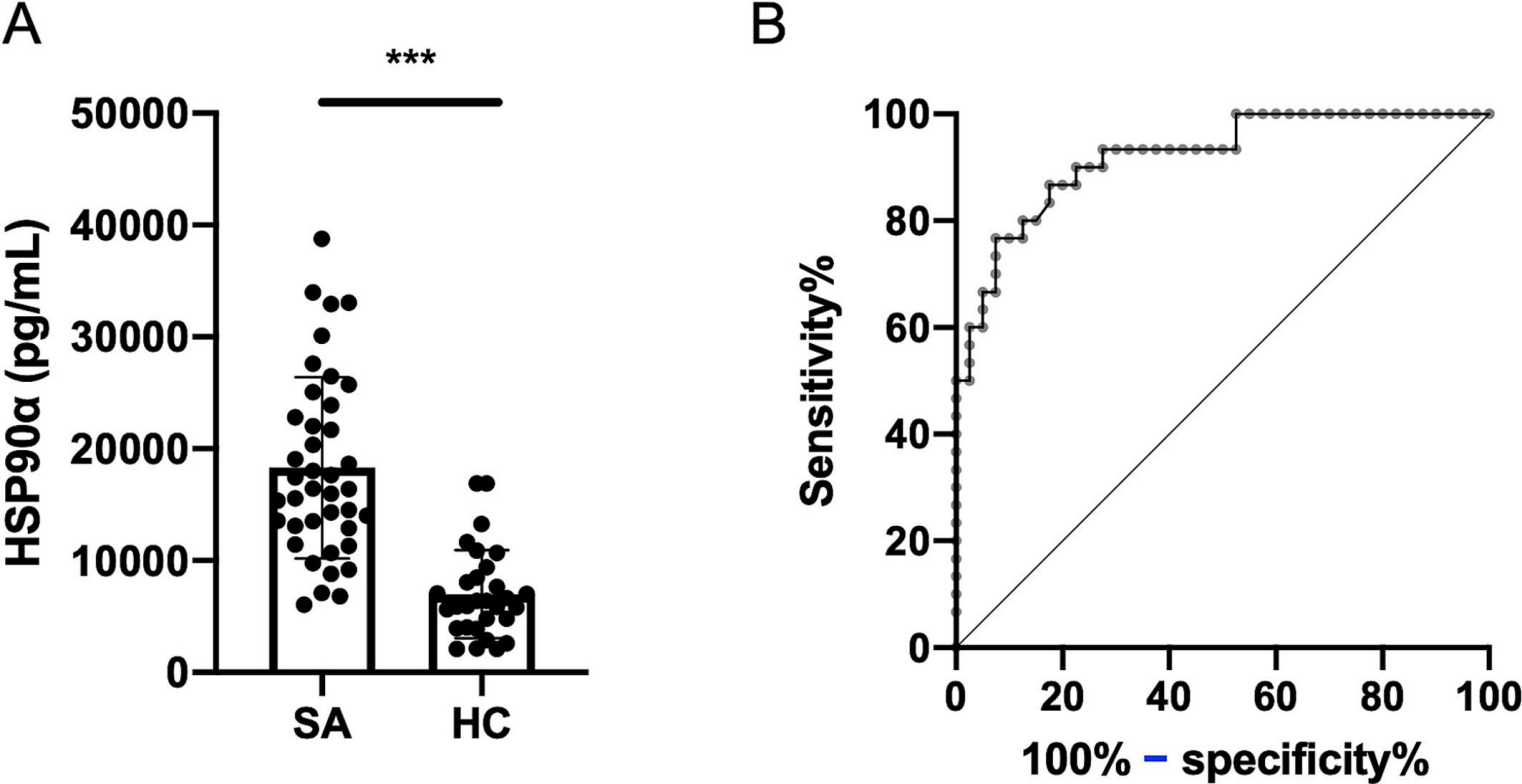
Serum HSP90α concentration. **(A)** Serum eHSP90α levels in sarcoidosis patients (SA) (*n* = 40) and healthy controls (HC) (*n* = 30). **(B)** ROC curve discriminating SA and HC with 86.7% sensitivity and 82.5% specificity. The AUC was 0.9196 (*p* < 0.0001).

Serum eHSP90α levels were significantly higher in sarcoidosis patients (18,300 ± 8,100 vs. 6,988 ± 3,930 pg./mL, *p* < 0.0001) (Figure 3A). ROC curve was generated to determine the diagnostic value of serum eHSP90α (Figure 3B). A cut-off value of 11,088 pg./mL allowed discrimination of sarcoidosis patients and healthy controls with 86.7% sensitivity and 82.5% specificity (area under the curve [AUC] 0.9196, *p* < 0.0001).

### Correlation between eHSP90α level and sarcoidosis disease activity

**Figure 4:**
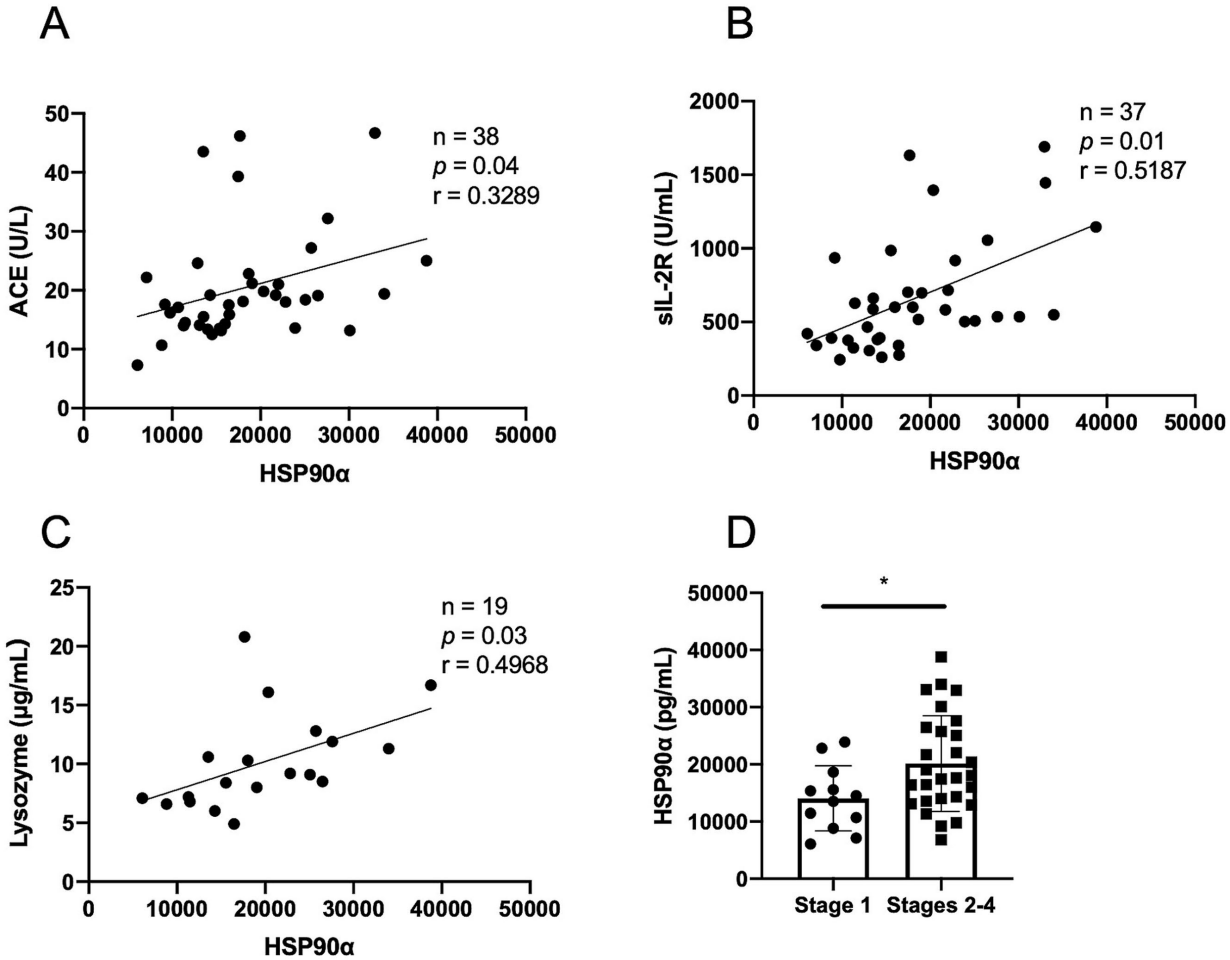
Association between serum HSP90α level and sarcoidosis disease activity. Correlation between serum eHSP90α and other biomarkers of sarcoidosis, including angiotensin-converting enzyme (ACE) **(A)**, soluble interleukin-2 receptor (sIL-2R) **(B)**, and lysozyme **(C)**. **(D)** Serum levels of HSP90α in stage 1 and stages 2–4 of sarcoidosis.

To determine whether serum eHSP90α reflects sarcoidosis disease activity, we examined the associations between eHSP90α level and other disease markers. Serum eHSP90α level in sarcoidosis patients was significantly correlated with several biomarkers of sarcoidosis, including ACE, sIL-2R, and lysozyme (Figures 4A–C).

When examining the eHSP90α associations in the chest stages of the disease (12), the serum eHSP90α level was found to be higher in patients at more advanced chest stages compared with patients at lower chest stages (14,035 ± 5,697 vs. 20,128 ± 8,366 pg./mL, *p* = 0.031) (Figure 4D). These data suggest that serum eHSP90α corresponds to sarcoidosis disease activity and severity.

### eHSP90α concentration in sarcoidosis lung

**Figure 5:**
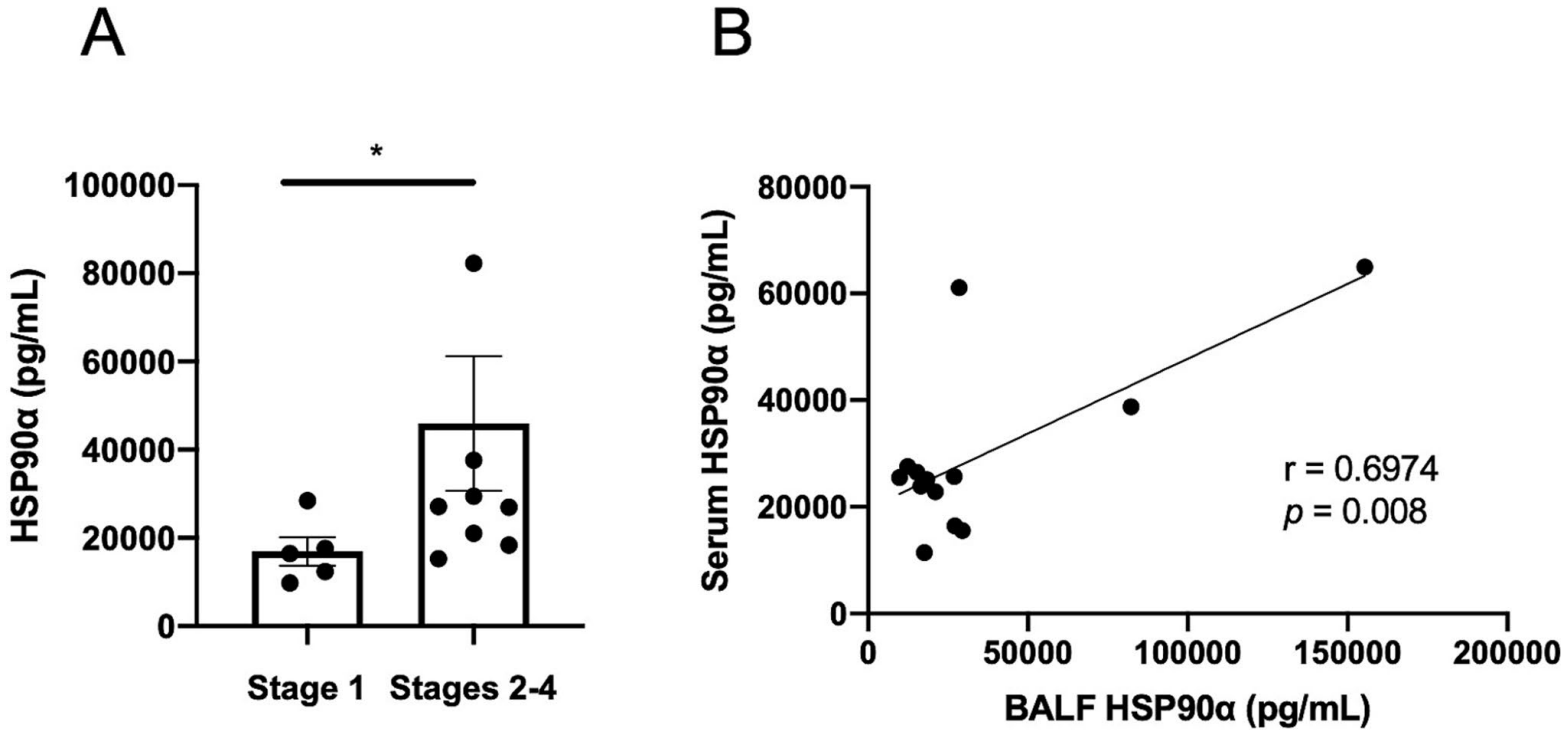
HSP90α levels in BALF of sarcoidosis patients. **(A)** BALF HSP90α concentration was elevated in patients in stages 2–4 compared with stage 1 patients. **(B)** BALF and serum HSP90α levels were significantly correlated in the same patients (r = 0.6974, *p* = 0.008).

The level of eHSP90α in BALF was determined as a measure of eHSP90α in the lung. HSP90α was detectable by ELISA in the BALF of sarcoidosis patients (35,595 ± 38,816 pg./mL, *n* = 14), and the HSP90α level in BALF was higher in severe patients compared with patients at lower chest stages (16,928 ± 7,173 vs. 45,966 ± 45,649 pg./mL, *p* = 0.042) (Figure 5A).

To examine the association between serum and lung HSP90α levels, we analyzed the correlation between serum and BALF HSP90α concentrations in the same patients (Figure 5B) and found a strong correlation of BALF and serum HSP90α (r = 0.6974, *p* = 0.008).

### eHSP90α secretion by macrophages

**Figure 6:**
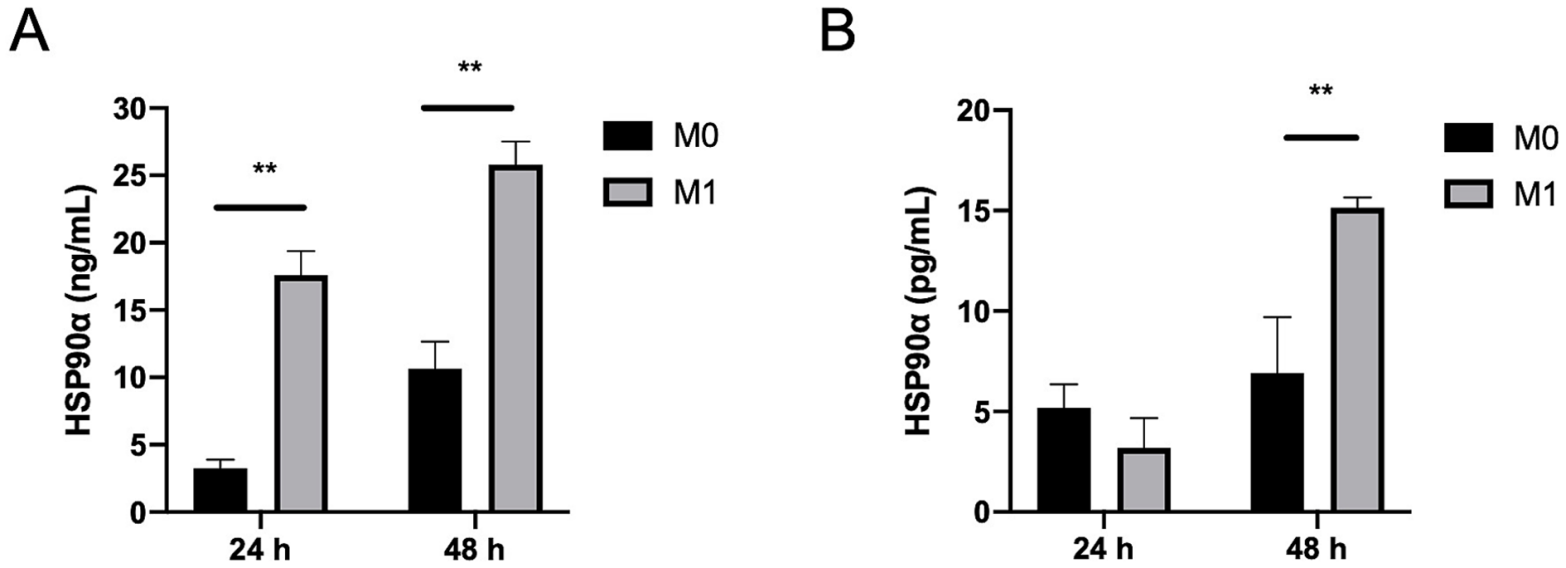
Secretion of extracellular HSP90α (eHSP90α) from macrophages. **(A)** THP1-macrophages were stimulated with or without M1 cytokine cocktail for 24 h or 48 h, after which the eHSP90α level in the supernatant was determined (*n* = 4). **(B)** Human MDMs were stimulated with or without M1 cytokine cocktail for 24 h or 48 h, after which the eHSP90α level in the supernatant was determined (*n* = 3).

Based on the results of staining of tissues from healthy and sarcoidosis lungs, we hypothesized that macrophages are a source of eHSP90α in the circulation and lungs. To examine the capacity of macrophages to produce eHSP90α, we differentiated THP1 monocytes into macrophages and polarized them into the M1 phenotype. Measurement of eHSP90α level in the supernatant demonstrated that macrophages can constitutively secrete HSP90α to the extracellular space, and cytokine stimulation can further promote HSP90α production (Figure 6A). Similar to cell lines, human MDMs can also produce HSP90α at steady state, and M1 macrophages are capable of secreting greater amounts of HSP90*α* (Figure 6B).

## Discussion

The pathogenesis of sarcoidosis is believed to start with the interaction between antigen-presenting cells and unidentified antigens, possibly infectious agents (e.g., *Propionibacterium acnes*, *Mycobacterium*), organic agents, and inorganic agents in genetically predisposed subjects (13, 14).

Innate immune cells, including alveolar macrophages, are activated through pattern recognition receptors and release proinflammatory and type 1 helper T (Th1)-skewing molecules such as IL-1, −6, −12, and − 18, tumor necrosis factor (TNF)-*α*, and IFN-*γ* (14, 15). These cytokines can promote the differentiation of CD4^+^ helper T cells into Th1 cells (16). Differentiated and activated Th1 cells also secrete these cytokines to alternatively activate macrophages, which can result in further promotion of inflammatory granulomatous signaling.

In *in vitro* systems, macrophages are functionally classified as M1 or M2, and stimulation assays can polarize/reprogram them into either phenotype. M1 macrophages exhibit antimicrobial activity in response to pathogens by releasing pro-inflammatory cytokines and chemokines such as TNF-α, IL-1β, IL-6, and CXCL10 (17, 18). By contrast, M2 macrophages (also called alternatively activated macrophages) can be induced by Th2-type inflammatory mediators and are characterized by an anti-inflammatory nature (17). During granuloma formation, M1 macrophages (also known as “classically activated” macrophages) are regarded as disease initiators (14). Our results indicated that HSP90α is highly expressed in epithelioid granulomas and that M1 macrophages can produce abundant eHSP90α compared with steady-state macrophages, which might support the hypothesis that HSP90α is contributing to the development and/or progression of sarcoidosis.

HSP90 is a molecular chaperone primarily involved in mediating the proper folding of proteins and correcting their localization, as well as regulating the disposal of incorrectly folded proteins (4). Proteins processed by HSP90 are referred to as “client proteins.” The interaction between HSP90 and a client protein is essential for normal biological processes and also plays a role in tumor survival, growth, and migration (19). HSP90 has two isoforms, HSP90α and HSP90β, which are encoded by identical cytosolic genes, with 86% homology (20). These two isoforms are from identical pools with different roles; the function of HSP90β seems to be limited to the intracellular form, whereas that of eHSP90α involves cellular responses to the microenvironment.

Recent studies have suggested that eHSP90α is an important immunomodulator due to its signaling functions (9) F5 peptides located in the linker of eHSP90 can bind to LRP1 on the cell membrane surface and transduce oncogenic, wound-healing, or inflammatory signals via phosphorylation of STAT3, ERK1/2, PI3K, and/or AKT1/2 (5–7). Therapeutic strategies targeting eHSP90 could thus regulate these signaling pathways without compromising the intracellular chaperone mechanism and therefore could represent a promising therapeutic approach in oncology and other fields in terms of target site accessibility and safety (21).

Secretion of eHSP90 in response to oxidative stress was first described in vascular smooth muscle cells but has also been reported to occur in tumor cells and fibroblasts (22–24). In the presence of various stressors, such as reactive oxygen species, hypoxia, UV radiation, or tissue injury, several-fold higher levels of eHsp90α protein have been detected in conditioned medium compared with resting cells without stimulation (7, 8, 22–24). Similar to reports regarding other types of cells, our study revealed that macrophages produce eHSP90α constitutively and that its production can be increased in response to cytokine stimulation. Other studies have reported that HSP90α is expressed in PBMCs and macrophages, and inhibition of HSP90α has been shown to suppress monocyte- and macrophage-derived inflammatory responses (25–28). High expression of HSP90α in lung macrophages and the production of eHSP90α by THP1-derived macrophages and primary macrophages observed in the present study are consistent with these reports and suggest that macrophages are a cellular source of eHSP90α.

Measurement of eHSP90α levels in the circulation has been reported as a predictive biomarker of lung cancer and pulmonary fibrosis progression (9). Furthermore, serum eHSP90α levels are increased in children with systemic inflammatory response syndrome compared with healthy children, and the level of eHSP90α has been associated with the development of multiple organ system failure (10). In the present study, serum eHSP90α levels were higher in patients with sarcoidosis compared with healthy controls and correlated with several markers reflective of sarcoidosis diseases activity. In addition, serum eHSP90α levels were high in advanced chest stages. Notably, eHSP90α secreted into the lungs was correlated with serum eHSP90α and reflected radiologic shadings. These results indicate that the serum eHSP90α level is a promising biomarker associated with sarcoidosis disease status.

In conclusion, HSP90α is highly expressed in lung macrophages. Various cell lines and primary macrophages secrete eHSP90α, particularly following cytokine stimulation. HSP90α levels in the serum and BALF are elevated in sarcoidosis patients, which could reflect disease activity. eHSP90α might therefore become a potential new biomarker of this disease, but this needs to be evaluated in large prospective cohorts. Furthermore, functional analysis of eHSP90α and identification of cells activated by the released eHSP90α will allow us to discuss eHSP90α as a potential specific therapeutic target for the disease.

## Data Availability

The original contributions presented in the study are included in the article/Supplementary material, further inquiries can be directed to the corresponding author.

## References

[1] Valeyre D Prasse A Nunes H Uzunhan Y Brillet P-Y Müller-Quernheim J. Sarcoidosis. Lancet. (2014) 383:1155–67. doi: 10.1016/S0140-6736(13)60680-7 24090799

[2] Vorselaars ADM Van Moorsel CHM Zanen P Ruven HJT Claessen AME Van Velzen-Blad H. ACE and sIL-2R correlate with lung function improvement in sarcoidosis during methotrexate therapy. Respir Med. (2015) 109:279–85. doi: 10.1016/j.rmed.2014.11.009, PMID: 25496652

[3] Miyoshi S Hamada H Kadowaki T Hamaguchi N Ito R Irifune K. Comparative evaluation of serum markers in pulmonary sarcoidosis. Chest. (2010) 137:1391–7. doi: 10.1378/chest.09-1975 20081103

[4] Hoter A El-Sabban M Naim H. The HSP90 family: structure, regulation, function, and implications in health and disease. IJMS. (2018) 19:2560. doi: 10.3390/ijms19092560, PMID: 30158430 PMC6164434

[5] Tsen F Bhatia A O’Brien K Cheng C-F Chen M Hay N. Extracellular heat shock protein 90 signals through subdomain II and the NPVY motif of LRP-1 receptor to Akt1 and Akt2: a circuit essential for promoting skin cell migration *in vitro* and wound healing *in vivo*. Mol Cell Biol. (2013) 33:4947–59. doi: 10.1128/MCB.00559-13, PMID: 24126057 PMC3889557

[6] Calderwood SK Khaleque MA Sawyer DB Ciocca DR. Heat shock proteins in Cancer: chaperones of tumorigenesis. Trends Biochem Sci. (2006) 31:164–72. doi: 10.1016/j.tibs.2006.01.006, PMID: 16483782

[7] Wong DS Jay DG. Emerging roles of extracellular Hsp90 in Cancer. Adv Cancer Res. (2016) 129:141–63. doi: 10.1016/bs.acr.2016.01.001, PMID: 26916004

[8] Bellaye P-S Shimbori C Yanagihara T Carlson DA Hughes P Upagupta C. Synergistic role of HSP90α and HSP90β to promote Myofibroblast persistence in lung fibrosis. Eur Respir J. (2018) 51:1700386. doi: 10.1183/13993003.00386-2017, PMID: 29386344

[9] Tanguy J Pommerolle L Garrido C Kolb M Bonniaud P Goirand F. Extracellular heat shock proteins as therapeutic targets and biomarkers in Fibrosing interstitial lung diseases. Int J Mol Sci. (2021) 22:9316. doi: 10.3390/ijms22179316, PMID: 34502225 PMC8430559

[10] Fitrolaki M-D Dimitriou H Venihaki M Katrinaki M Ilia S Briassoulis G. Increased extracellular heat shock protein 90α in severe Sepsis and SIRS associated with multiple organ failure and related to acute inflammatory-metabolic stress response in children. Medicine. (2016) 95:e4651. doi: 10.1097/MD.0000000000004651, PMID: 27583886 PMC5008570

[11] American Thoracic Society. Statement on sarcoidosis. Respir Crit Care Med. (1999) 160:736–55. doi: 10.1164/ajrccm.160.2.ats4-99, PMID: 10430755

[12] Scadding JG. Prognosis of intrathoracic sarcoidosis in England. BMJ. (1961) 2:1165–72. doi: 10.1136/bmj.2.5261.1165 14497750 PMC1970202

[13] Cinetto F Agostini C. Advances in understanding the immunopathology of sarcoidosis and implications on therapy. Expert Rev Clin Immunol. (2016) 12:973–88. doi: 10.1080/1744666X.2016.1181541, PMID: 27101234

[14] Drent M Crouser ED Grunewald J. Challenges of sarcoidosis and its management. N Engl J Med. (2021) 385:1018–32. doi: 10.1056/NEJMra2101555, PMID: 34496176

[15] Rastogi R Du W Ju D Pirockinaite G Liu Y Nunez G. Dysregulation of P38 and MKP-1 in response to NOD1/TLR4 stimulation in sarcoid Bronchoalveolar cells. Am J Respir Crit Care Med. (2011) 183:500–10. doi: 10.1164/rccm.201005-0792OC, PMID: 20851927 PMC5450927

[16] Darlington P Kullberg S Eklund A Grunewald J. Subpopulations of cells from Bronchoalveolar lavage can predict prognosis in sarcoidosis. Eur Respir J. (2020) 55:1901450. doi: 10.1183/13993003.01450-2019, PMID: 31439687 PMC6991162

[17] Shapouri-Moghaddam A Mohammadian S Vazini H Taghadosi M Esmaeili S Mardani F. Macrophage plasticity, polarization, and function in health and disease. J Cell Physiol. (2018) 233:6425–40. doi: 10.1002/jcp.26429, PMID: 29319160 PMC13482560

[18] Mantovani A Sica A Sozzani S Allavena P Vecchi A Locati M. The chemokine system in diverse forms of macrophage activation and polarization. Trends Immunol. (2004) 25:677–86. doi: 10.1016/j.it.2004.09.015, PMID: 15530839

[19] Trepel J Mollapour M Giaccone G Neckers L. Targeting the dynamic HSP90 complex in Cancer. Nat Rev Cancer. (2010) 10:537–49. doi: 10.1038/nrc2887, PMID: 20651736 PMC6778733

[20] Li W Tsen F Sahu D Bhatia A Chen M Multhoff G. Extracellular Hsp90 (eHsp90) as the actual target in clinical trials: intentionally or unintentionally. Int Rev Cell Mol Biol. (2013) 303:203–35. doi: 10.1016/B978-0-12-407697-6.00005-2, PMID: 23445811 PMC4023563

[21] Jay D Luo Y Li W. Extracellular heat shock Protein-90 (eHsp90): everything you need to know. Biomol Ther. (2022) 12:911. doi: 10.3390/biom12070911, PMID: 35883467 PMC9313274

[22] Liao D-F Jin Z-G Baas AS Daum G Gygi SP Aebersold R. Purification and identification of secreted oxidative stress-induced factors from vascular smooth muscle cells. J Biol Chem. (2000) 275:189–96. doi: 10.1074/jbc.275.1.189, PMID: 10617604

[23] Cheng C-F Fan J Fedesco M Guan S Li Y Bandyopadhyay B. Transforming growth factor α (TGFα)-stimulated secretion of HSP90α: using the receptor LRP-1/CD91 to promote human skin cell migration against a TGFβ-rich environment during wound healing. Mol Cell Biol. (2008) 28:3344–58. doi: 10.1128/MCB.01287-07 18332123 PMC2423165

[24] Li W Li Y Guan S Fan J Cheng C-F Bright AM. Extracellular heat shock protein-90α: linking hypoxia to skin cell motility and wound healing. EMBO J. (2007) 26:1221–33. doi: 10.1038/sj.emboj.7601579, PMID: 17304217 PMC1817627

[25] Qiao Y Wang C Kou J Wang L Han D Huo D. MicroRNA-23a suppresses the apoptosis of inflammatory macrophages and foam cells in Atherogenesis by targeting HSP90. Gene. (2020) 729:144319. doi: 10.1016/j.gene.2019.144319, PMID: 31884108

[26] Bzowska M Nogieć A Bania K Zygmunt M Zarębski M Dobrucki J. Involvement of cell surface 90 kDa heat shock protein (HSP90) in pattern recognition by human monocyte-derived macrophages. J Leukoc Biol. (2017) 102:763–74. doi: 10.1189/jlb.2MA0117-019R, PMID: 28550115 PMC5557637

[27] Choudhury A Bullock D Lim A Argemi J Orning P Lien E. Inhibition of HSP90 and activation of HSF1 diminish macrophage NLRP3 Inflammasome activity in alcohol-associated liver injury. Alcohol Clin Exp Res. (2020) 44:1300–11. doi: 10.1111/acer.14338, PMID: 32282939 PMC7328660

[28] Zhou Z Li X Qian Y Liu C Huang X Fu M. Heat shock protein 90 inhibitors suppress Pyroptosis in THP-1 cells. Biochem J. (2020) 477:3923–34. doi: 10.1042/BCJ20200351, PMID: 32497199 PMC7773284

