# Supplementary material for: Upregulation of HSP90α in the lungs and circulation in sarcoidosis
Source: Front Med (Lausanne). 2025 Jan 15;12:1532437. doi: 10.3389/fmed.2025.1532437 (PMC11774731; doi:10.3389/fmed.2025.1532437)
Supplement: Supplementary file 1 [file Presentation_1.pptx]

## Slide 1
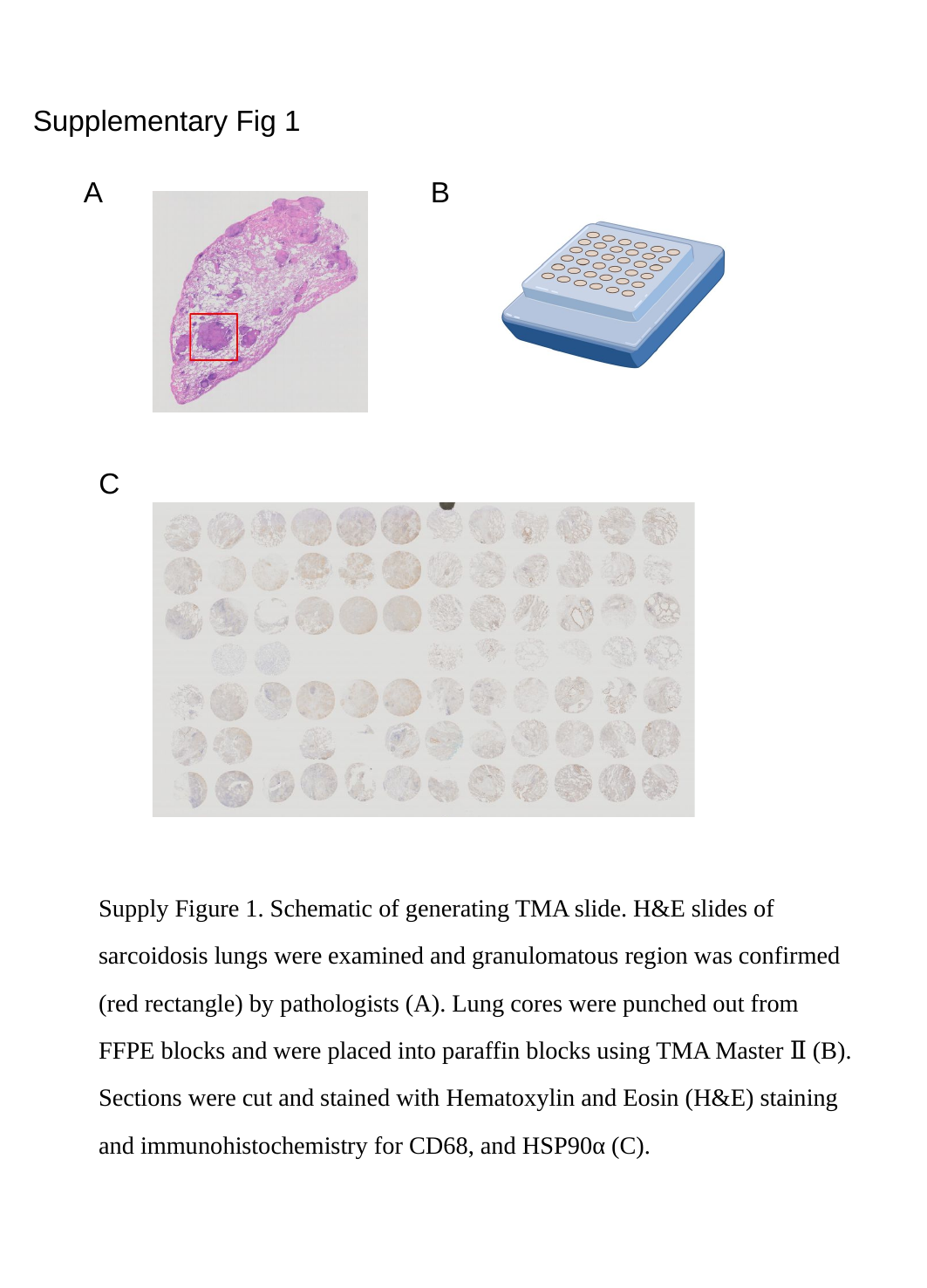

# Supplementary Fig 1
A
B
C
Supply Figure 1. Schematic of generating TMA slide. H&E slides of sarcoidosis lungs were examined and granulomatous region was confirmed (red rectangle) by pathologists (A). Lung cores were punched out from FFPE blocks and were placed into paraffin blocks using TMA Master Ⅱ (B). Sections were cut and stained with Hematoxylin and Eosin (H&E) staining and immunohistochemistry for CD68, and HSP90α (C).
